# Iron Oxide Silica Derived from Sol-Gel Synthesis

**DOI:** 10.3390/ma4020448

**Published:** 2011-02-17

**Authors:** Adi Darmawan, Simon Smart, Anne Julbe, João Carlos Diniz da Costa

**Affiliations:** 1 FIMLab—Films and Inorganic Membrane Laboratory, School of Chemical Engineering, The University of Queensland, Brisbane Qld 4072, Australia; E-Mails: a.darmawan@uq.edu.au (A.D.); s.smart@uq.edu.au (S.S.); 2 Institut Europeen des Membranes, Universite Montpellier 2, Place Eugene Bataillon 34095, Montpellier, cedex 5, France; E-Mail: Anne.Julbe@iemm.univ-montp2.fr

**Keywords:** iron oxide, silica network, molecular sieve structures

## Abstract

In this work we investigate the effect of iron oxide embedded in silica matrices as a function of Fe/Si molar ratio and sol pH. To achieve homogeneous dispersion of iron oxide particles, iron nitrate nonahydrate was dissolved in hydrogen peroxide and was mixed with tetraethyl orthosilicate and ethanol in a sol-gel synthesis method. Increasing the calcination temperature led to a reduction in surface area, although the average pore radius remained almost constant at about 10 Å, independent of the Fe/Si molar ratio or sol pH. Hence, the densification of the matrix was accompanied by similar reduction in pore volume. However, calcination at 700 °C resulted in samples with similar surface area though the iron oxide content increased from 5% to 50% Fe/Si molar ratio. As metal oxide particles have lower surface area than polymeric silica structures, these results strongly suggest that the iron oxides opposed the silica structure collapse. The effect of sol pH was found to be less significant than the Fe/Si molar ratio in the formation of molecular sieve structures derived from iron oxide silica.

## 1. Introduction

The sol-gel process is a very flexible route for producing molecular sieving structures with pore radii in the region of ~10 Å or lower. These structures are attractive in thin film applications such as gas separation membranes and sensors. For these applications, tailoring the silica architecture is crucial, as the ideal pore size will hinder the passage of large molecules, but allow access to smaller molecules. These molecular sieving structures are often produced from the synthesis of polymeric silicate gels involving the hydrolysis of monomeric tetrafunctional alkoxide precursors and a mineral acid (e.g., HCl) or a base (e.g., NH_3_), as catalysts [1]. Here, the pore size is controlled by the sol-gel synthesis conditions. Of particular importance is the water to silica molar ratio, as reasonably low H_2_O/Si molar ratio (<10) tends to inhibit the condensation reactions, thus favoring the formation of silanol groups (Si-OH) and pore sizes below 10 Å [2]. Conversely, a high H_2_O/Si molar ratio provides the sol-gel synthesis with excess water, thus benefiting the condensation reaction and the formation of siloxane bridges (Si-O-Si) and larger pore sizes [3].

A further important technique to tailor the pore size is to add organic templating agents during the sol-gel process [4]. In this process, organic templates trapped in the gel structure are burnt off at high temperature under oxidizing conditions, thus producing a cavity with similar dimensions to that of the template molecule [5]. Several organic templates have been employed by researchers to form microstructures [6] including methyltriethoxysilane [7], octyl-, dodecyl- or octadecyltriethoxysilane [8] and methacryloxypropyl groups [9]. Using this technique, researchers also prepared molecular sieving architectures by calcining templated sol-gels in inert atmospheres using methyltriethoxysilane [10], surfactants [11], as well as natural organic products such as sucrose [12] and starch [13].

Recently, there has been limited activity of incorporating metals and metal oxides in the sol-gel process to successfully produce molecular sieve silica. Metal oxides or metal doped silica sols were prepared through the hydrolysis and condensation of tetraethylorthosilicate (TEOS) in ethanol and hydrogen peroxide (H_2_O_2_) with hydrated cobalt and nickel nitrate salts or niobia [14,15,16,17,18]. It is well documented in the literature that the fine control of the silica architecture and pore size tuning is possible using sol-gel processes. In this work, we investigate the effect of incorporating iron oxide into molecular sieving silica. Important parameters considered in this study are the silica to water molar ratio, iron oxide to silica molar ratio, sol pH, functional groups, and their influence in the formation of molecular sieving structures.

## 2. Results and Discussion

**Figure 1:**
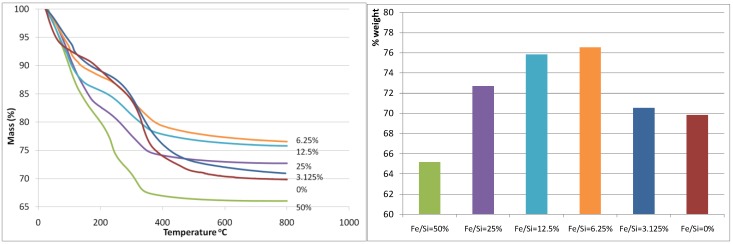
(**a-left**) Mass (%) curves (calculated relative to mass at room temperature); (**b-right**) total weight loss. Both are as a function of the Fe/Si molar ratio.

Figure 1(a) and Figure 1(b) display the thermogravemetric analysis (TGA) weight loss curve and the total weight loss of the iron oxide silica xerogels, respectively. The total weight loss was almost unaltered when the Fe/Si molar ratio increased from 0 to 3.125%, however there is a marked decrease in weight loss at Fe/Si 6.25% corresponding to the minimum weight loss observed for all samples. From there on, the total weight loss increased exponentially each time that the Fe/Si molar ratio increased. These results strongly suggest that the addition of iron affects the sol-gel processes. These trends can also be observed as each curve evolves through the heat treatment in air as shown in Figure 1(a). For instance, the blank and 3.125% samples show similar TGA curves, while the remaining samples from 6.25% to 50% show similar trends to each other but different to the blank sample. In terms of TGA behavior, the 3.125% iron content sample is behaving like the blank silica sample, so the small addition of iron is not significant. On the other hand, for samples 6.25% and above, it appears the iron content plays a major role. This is increasingly evident as the individual weight loss peaks are analyzed across the samples. From 80–120 °C, the weight loss is generally caused by the loss of free (not chemically bound) H_2_O and ethanol molecules, and there is a sharp increase in weight loss that is again associated with the increase of Fe/Si molar ratio, suggesting that an increasing iron content results in increased H_2_O within the uncalcined xerogel matrix.

**Figure 2:**
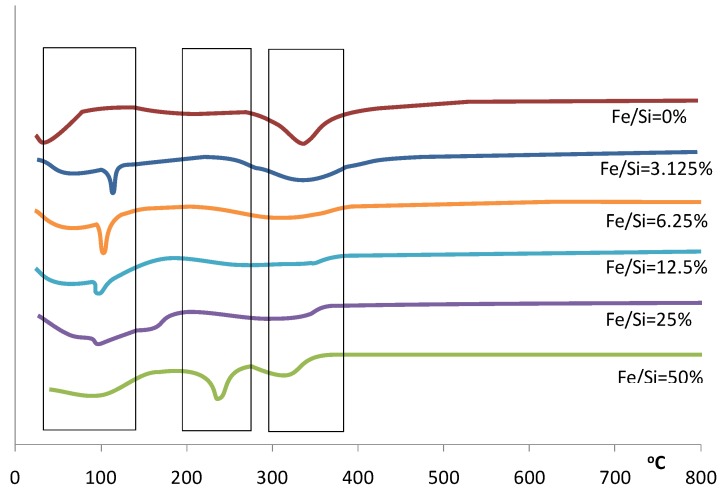
Differential of the TGA for samples prepared with varying Fe/Si molar ratios.

This trend continues over the next weight loss peaks between 120–400 °C, whereby increasing weight losses coincide with increasing iron content. This is especially true for the 50% Fe/Si molar ratio sample. For pure silica sol-gels analyzed by TGA within this temperature range, the weight losses are generally associated with the condensation reactions due to heat treatment, resulting in the production of water and ethanol, which evaporates from the silica matrix. However, the decomposition of iron nitrate nonahydrate should lead to the formation of a coordinated Fe(OH)_6_ octahedral structure [19], where Fe is surrounded by six oxygen atoms corresponding to OH groups or H_2_O molecules. Due to the fact that a reasonable amount of water agglomerates around iron, increasing the iron content in the sol-gel will likewise significantly increase the amount of water in the sample. The additional weight loss seen for increasing iron content is therefore assigned to the removal of additional water, as well as solvents (ethanol) and organics (R groups from TEOS). Furthermore, the nitrate decomposition, related to the removal of HNO_3_ from the hydrated salt [19] and eventual NO and NO_2_ formation which may take place around 230–240 °C, must also be considered. This decomposition is observed in the dTGA differential analysis in Figure 2 for the 50% Fe/Si molar ratio sample.

**Figure 3:**
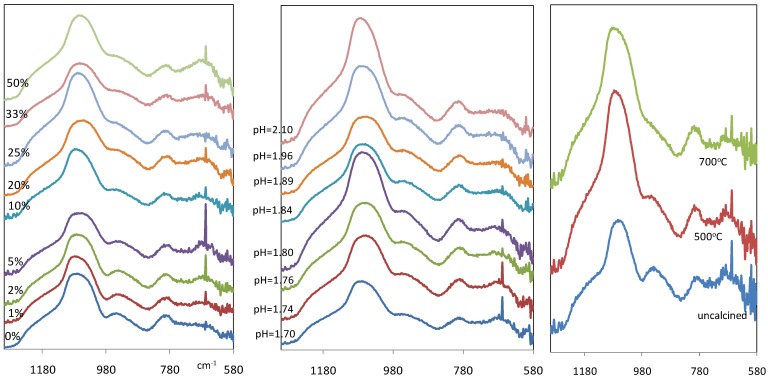
IR spectra of iron oxide silica xerogel samples as a function of (**a-left**) Fe/Si molar ratio; (**b-middle**) pH for 10% Fe/Si molar ratio sample; and (**c-right**) calcinations temperature for 10% Fe/Si molar ratio sample.

Figure 3(a) and Figure 3(b) show the IR spectra of xerogel samples between wave number 1300 and 580 cm^−1^. It is observed that regardless of the Fe/Si molar ratio variation, or pH variation, the IR spectra of the xerogel samples remained almost constant. These results suggest that no significant variation of silica functional groups was attained by introduction of iron and pH changes. These results concur with previous literature in the sense that the H_2_O/Si molar ratio was kept constant in the sol for all samples [3]. Hence, the formation of the silanol and siloxane groups was still governed by the silica hydrolysis and condensation reactions.

However, a notable difference in Figure 3a and Figure 3b is a new band which is observed at 667 cm^−1^. This band becomes more prominent as the Fe/Si molar ratio increases towards 50%, and it was not found in the pure silica xerogel. Since there are no corresponding bands in the infrared spectra of silica in this region, this new band is most probably associated with Fe-O stretching vibration, a region generally associated with metal oxides [17]. The other bands observed are conventionally found in silica materials as reported elsewhere [20,21,22]. This includes the bands at 800 cm^−1^ and 1076 cm^−1^ with a shoulder near 1200 cm^−1^ corresponding to different modes of siloxane stretching bonds (Si-O-Si): symmetric and asymmetric, respectively. The shoulder in the region of 960 cm^−1^ is assigned to the stretching vibration of Si-OH.

Figure 3(c) compares the IR spectra of iron oxide silica xerogel samples before and after calcination at 500 and 700 °C. The band at ~960 cm^−1^ associated with silanol groups, is weakened gradually with heat treatment, changing from a distinct peak for the uncalcined sample to a broad shoulder at 500 °C. In addition, the intensity of the peak around 1080 cm^−1^, assigned to siloxane bridges, increased. These changes are attributed to condensation reactions, and supports the view that polycondesation allows for the transformation of silanols to siloxane bridges. As heat treatment progresses, the band at 1067 cm^−1^ for the uncalcined sample shifts to 1076 and 1083 cm^−1^ at 500 and 700 °C, respectively. This is interpreted as changes in the xerogel network caused by irreversible shrinkage in the silica polymeric bonding derived from heat treatment. In addition, it is observed that there are no bands assigned to Fe-O-Si bonds at ~490 cm^−1^ region as reported elsewhere [23,24]. These results clearly suggest that calcination results in the formation of a mixed matrix of polymeric silica and iron oxide particles.

**Figure 4:**
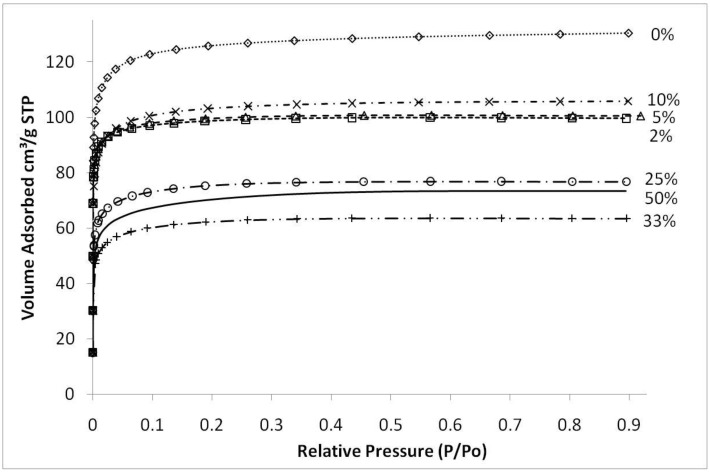
Nitrogen sorption isotherms of samples with varying Fe/Si molar ratio for sample calcined 500 °C.

Figure 4 shows typical nitrogen adsorption isotherms of iron oxide silica xerogels calcined at 500 °C. These isotherms are type 1, characteristic of microporous materials. These results suggest that that sol-gel method used in this work essentially produced molecular sieves of similar structure, independently of the content of iron oxide added to the silica matrix. The average pore radius remained similar for all samples at around 10 Å (±0.5 Å) though pore volumes and surface areas varied depending upon the preceding sol-gel synthesis conditions and calcination temperatures. The only exception was the sample synthesized with pH 2.1, close to the precipitation point of the sol-gel, which showed an average pore radius of 11.7 Å (±0.5 Å).

**Figure 5:**
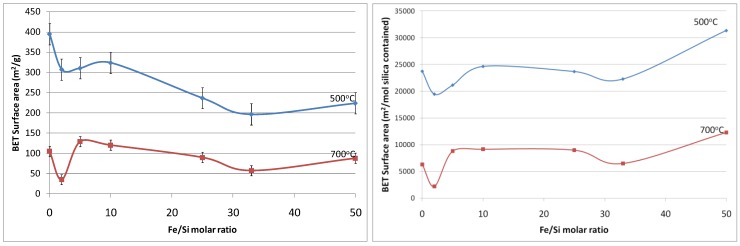
(**a-left**) Surface area of samples; and (**b-right**) surface area per mole of silica, as a function of Fe/Si molar ratio.

Figure 5a shows the resultant BET (Brunauer, Emmett and Teller) surface area as a function of Fe/Si molar ratio. First of all, the effect of temperature calcination is consistent with conventional pure silica calcination. That is that increasing the calcination temperature densifies the silica matrix. The initial changes from the blank sample to 2% Fe/Si molar ratio resulted in a significant reduction in surface area of 33% and ~50% at 500 and 700 °C, respectively. From there on, surface areas tended to increase and then slightly decrease as the iron oxide content increases. Similar trends were also observed for changes of pore volume *versus* Fe/Si molar ratio, as the average pore radius remained constant around 10 Å (±0.5 Å). The initial subtle changes for the 2% Fe/Si sample seems to be an anomaly, though it could be correlated to the TGA trends displayed in Figure 1. At low Fe/Si ratio, the iron oxide effect is not significant, and in this case does not oppose the densification of the silica matrix. Similar to the TGA results, increasing the iron oxide content further provides beneficial structural stability. This point is clearly observed in Figure 5b. In principle the surface area and microporous pore volume should reduce as the amount of iron oxide particles, which generally have a low surface area as compared to polymeric silica, increases and the silica content decreases per gram of sample. This is not the case, thus suggesting that iron oxide particles were homogeneously dispersed in the silica matrix. In addition, the iron oxides opposed a greater collapse of the silica matrix, particularly as the Fe content increased from 33 to 50%, as the surface areas per mole of silica increased.

**Figure 6:**
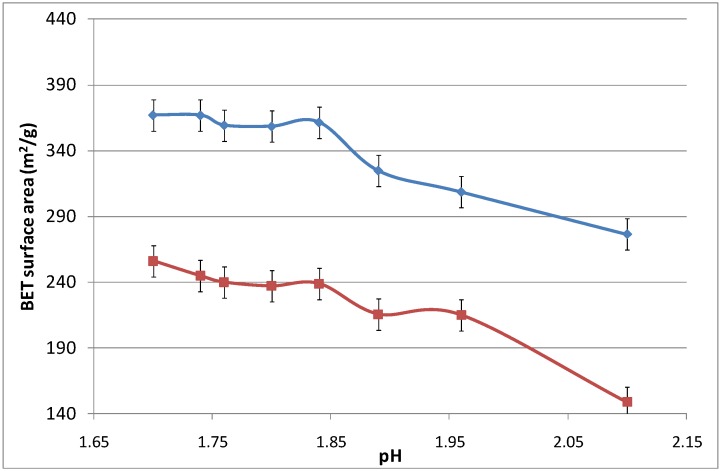
Surface area of samples as a function of pH.

Figure 6 displays the surface area as a function of the pH, which was modified by adding an aqueous ammonia solution. Again, increasing the calcination temperature from 500 to 700 °C led to the densification of the iron oxide silica matrix. The surface area did not change significantly as the pH increased from 1.7 to 1.825, while slightly reducing the surface area from thereon. For pH values above 2.10, the sol-gel agglomerated and precipitated, and was no longer considered in this work as it was no longer in polymeric form.

## 3. Experimental Section

The iron oxide silica sol was prepared similar to metal oxide silica sol methods described elsewhere [17], the major difference being the use of iron oxides instead of nickel or cobalt oxides. Briefly, iron oxide sols were synthesized by the hydrolysis and condensation of tetraethyl orthosilicate (TEOS) in ethanol and 30% aqueous H_2_O_2_ with iron nitrate nonahydrate (Fe(NO_3_)_3_.9H_2_O). An initial molar ratio of 255 EtOH: 4 TEOS: x Fe(NO_3_)_3_.9H_2_O: 9H_2_O_2_: 40H_2_O was mixed and vigorously stirred for 3 hours in an ice-cooled bath. The number of moles of iron nitrate nonahydrate, x, was varied between 0–2 and the pH was adjusted with aqueous ammonia to determine the synthesis effects on the silica structure. The molar ratio is defined as the number of moles of Fe species divided by the number of moles of Si species. It should be noted that the water of crystallization was taken into account when calculating the water to silica molar ratio. A blank silica sample (x = 0) having no iron oxide was also prepared for comparison with the iron oxide silica.

Sol samples were dried in a temperature-controlled oven at 60 °C at atmospheric conditions to form a xerogel. The xerogel samples were crushed finely and calcined at ramp rates 1.5 °C min^−1^ to 500 °C and 700 °C in an oxidizing (air) atmosphere with a 3 hour holding time at the desired temperature. Nitrogen adsorption was studied at 77 K using a Micromeritics Tristar 3000 to determine the BET surface area. The samples were initially degassed for 24 h to pressures of ~2 Pa at 200 °C. Fourier transform infrared analysis (FTIR) was carried out on a Shimadzu IRAffinity-1 with a Pike MIRacle ATR attachment. Spectra were taken over a wavelength range of 1300–580 cm^−1^. Thermogravetric analysis (TGA) was performed using a Shimadzu TGA-50. Xerogel powders were heated at 2.0 °C min^−1 ^up to 800 °C with an air flow rate of 80 mL min^−1^.

## 4. Conclusions

The findings of this work clearly indicate that the content of iron oxide in the silica matrix has a significant effect on the resultant material matrix. Large weight losses were associated with OH groups or H_2_O molecules derived from the decomposition of iron nitrate nonahydrate in addition to solvents (ethanol) and organics (R groups from TEOS). Furthermore, nitrate decomposition under heat treatment led to NO and NO_2_ formation, further contributing to weight losses. In other words, increasing the Fe/Si molar ratio similarly increases the weight losses. No observable changes in the silica functional groups were found, thus suggesting no significant variation of silica functional group formation as a function of iron oxide content or sol pH.

The average pore radius remained almost constant in the region of 10 Å, indicating that surface areas and pore volumes varied proportionally with the calcination temperature, though resulting in the densification of the material matrix. However, calcination at 700 °C resulted in samples with similar surface area as the iron oxide content increased from 5% to 50% Fe/Si molar ratio. This result is counterintuitive as the increase in iron oxide content should decrease the total surface area. As metal oxide particles have low surface area than polymeric silica structures, these results strongly suggest that the iron oxides played a beneficial role in opposing the silica structure collapse.
